# Phenolic acids in fermented foods: microbial biotransformation, antioxidant mechanisms, and functional health implications

**DOI:** 10.3389/fmolb.2025.1678673

**Published:** 2025-10-21

**Authors:** Anand Kumar, S. Saranyadevi, Selva Kumar Thirumalaisamy, Tharindu Trishan Dapana Durage, Swapnil Ganesh Jaiswal, Digambar Kavitake, Shuai Wei

**Affiliations:** ^1^ College of Food Science and Technology, Guangdong Ocean University, Guangdong Provincial Key Laboratory of Aquatic Products Processing and Safety, Guangdong Province Engineering Laboratory for Marine Biological Products, Guangdong Provincial Engineering Technology Research Center of Seafood, Key Laboratory of Advanced Processing of Aquatic Product of Guangdong Higher Education Institution, Zhanjiang, China; ^2^ Department of Biotechnology, Faculty of Engineering (FoE), Karpagam Academy of Higher Education, Coimbatore, Tamil Nadu, India; ^3^ Vel Tech Rangarajan Dr. Sagunthala R&D Institute of Science and Technology, Chennai, Tamil Nadu, India; ^4^ School of Nutrition and Food Sciences, Louisiana State University Agricultural Center, Baton Rouge, LA, United States; ^5^ Food Engineering Laboratory, Department of Agricultural Engineering Maharashtra Institute of Technology Aurangabad, Aurangabad, Maharashtra, India; ^6^ Department of Food Science and Technology, Pondicherry University, Puducherry, India; ^7^ Biochemistry Division, National Institute of Nutrition (ICMR), Hyderabad, Telangana, India

**Keywords:** phenolic acids, fermentation, health benefits, microbial biotransformation, antioxidants, functional foods

## Abstract

Phenolic acids, a heterogeneous group of plant polyphenols that play a significant role in health due to their antioxidant, anti-inflammatory, and disease-modulating activities. Historically, fermentation has been recognized as a versatile biotechnology for increasing the bioavailability and efficacy of phenolic acids in foods. During microbial fermentation, indigenous and bound phenolics are subjected to extensive biotransformation by lactic acid bacteria, yeasts, and other functional microbes. These microorganisms synthesize hydrolytic and oxidative enzymes, including esterase, decarboxylase, and phenolic acid reductase, which release and alter phenolic acids, such as ferulic, p-coumaric, caffeic, and gallic acids, from plant cell wall matrices. Microbial conversion increases solubility, changes structural components, and enhances antioxidant capacity. Mechanistically, phenolic acids exhibit potent radical scavenging, metal-chelating, and singlet oxygen-quenching activities that play a significant part in reducing oxidative stress and redox homeostasis. Structure-activity relationships demonstrate that hydroxylation and methoxylation patterns have a critical impact on antioxidant strength. Additionally, phenolic acids involve numerous molecular methods, such as the activation of Nrf2–ARE, suppression of NF-κB, followed by the restoration of gut barrier integrity results in the anti-inflammatory, neuro-, cardio-protective, and immunomodulatory. The coactive interactions within phenolic acids, bacterial metabolites like short-chain fatty acids (SCFAs), as well as the components of the food matrix further strengthen their biological activity. Our review highlights the critical study of bacterial biotransformation of phenolic acids during fermentation, explicates their antioxidant mechanisms, and highpoints their emerging importance for functional health. Insights into these interrelationships are vital for the development of functional fermented foods, which enhance the therapeutic effect of managing chronic diseases and promote overall health.

## 1 Introduction

Over the past years, people have been eating fermented foods because they are higher in nutrients, have a longer shelf life, and have better flavors because of microbial activity (Marco et al., 2017). From cereals and legumes to dairy and vegetables, fermentation has played a crucial role in turning a variety of food sources into global dietary staples (J. A. Adebo et al., 2022; Sharma et al., 2020). Fermentation’s capacity to dramatically increase food’s antioxidant content is among its most appealing health benefits. Oxidative stress plays a significant role in the causes of chronic diseases such as cancer, diabetes, cardiovascular disease, and so on, which signify serious challenges to the world. The disproportion between antioxidant defense and reactive oxygen species (ROS) results in oxidative stress that damages cells and affects the body’s normal physiological functions. Nowadays, dietary actions to reduce oxidative stress, particularly via the intake of food with higher antioxidants, have gained attention. Most of the fermented foods that have crucial levels of phenolic acids, a class of plant-derived polyphenols, have stronger antioxidant characteristics (Saud et al., 2024). An enhanced bioavailability and biologically active form of phenolic acids were obtained during the fermentation process, which converts crucial plant substances into absorbable forms. Further, these substances prevent tissue damage caused by oxidative stress. Phenolic acid is found to be the most significant class of dietary polyphenols that is well-known for its better antioxidant qualities and related health benefits. Plant-based substances, including fruits, vegetables, and so on, where bacterial activity enhances their bioavailability. The most common ingredients, like cereals and bran, have been linked with cardiovascular and neurological impacts that show a potent capability to scavenge free radicals (Ouamnina et al., 2024). Caffeic acid is present in coffee and other fermented vegetables, which has anti-inflammatory and antioxidant features leading to better health (Alam et al., 2022).

These types of phenolic acids play a vital part in the deterrence and management of chronic illnesses like oxidative stress due to their scavenging activity of reactive oxygen species (ROS) and modulate cell signalling pathways. Although natural phenol-based substances have better antioxidant activities, it will be a challenging task to utilize them because of their bioavailability. Moreover, phenolic acids are less frequently absorbed in the gastrointestinal tract due to their high molecular size and low water solubility when disbursed in their natural state. The gut microbes and enzymes present in the intestine metabolize a huge variety of phenolic substances, by altering their biological activity and lowering their efficacy (De Almeida et al., 2022). The absorption and liberation of biologically active substances may be further incumbered through the existence of dietary fibers, cell walls, and other food matrix elements. Due to this property, only a minor portion of phenolic acid is consumed into the systemic-based circulation. Numerous methods have been determined to enhance the bioavailability of phenolic acids, like fermentation, processing of food, and encapsulation. Fermentation is particularly well-known for its capability to break down complex chemicals and yield them in a more accessible form. To exploit phenolics’ therapeutic potential in the deterrence and treatment of chronic disorders, though, a study is still undergoing to enhance their bioavailability (Das et al., 2023). A study showed that fermented foods like sourdough, tempeh, and fermented cereals have better concentrations of active phenolic acids than their unfermented foods (Dissanayake et al., 2025). Consequently, fermentation plays a significant part in optimizing the therapeutic potential of dietary phenolics, especially in treating oxidative stress and managing chronic diseases, in addition to preserving and improving the nutritional content of foods. Our review work aims to evaluate how fermented foods high in phenolic acid can reduce oxidative stress and how they might be utilized to prevent and treat chronic illnesses.

## 2 Common sources of phenolic acids in food

In the case of food sector, bacterial fermentation is most widely employed. It can produce a variety of secondary metabolites, including phenolic acids, in addition to the primary fermentation products, such as ethanol or lactic acid. For instance, higher amounts of hydroxybenzoic acids result in a 1.5–2.0-fold rise in the overall number of phenolic acids following wine fermentation (Tian et al., 2009). Phenolic acids are added to wheat bread doughs, and the quantity and composition of phenolic acids determine the dough’s quality and characteristics. While the amount of other phenolic acids, such as caffeic, sinapic, p-hydroxybenzoic, and gallic acids, is significantly lower, L. plantarum strains LB126, 29DAN, and 98A can enrich the doughs in ferulic acid up to 400 μg/g DW. When R. oryzae RCK2012 is employed in wheat fermentation, the product might be enriched with vanillic and p-hydroxybenzoic acids (Zhang et al., 2022a).

Phenolic acids in cereals are found in both free and bound forms, with the majority primarily existing in the bound state. Free phenolic acids are located on the surface of cereal grains and can be easily extracted using aqueous solvents, making them more readily bioaccessible. Conversely, bound phenolic acids are covalently attached to cell wall components such as lignin, cellulose, and arabinoxylans, especially within the bran layer. This structural attachment greatly restricts their release and absorption in the gastrointestinal tract. The most prevalent phenolic acid in cereals like wheat, corn, and rice is ferulic acid, which is normally present in a bound state and must be hydrolysed by enzymes or microbes in order to be released (Horvat et al., 2020). The effects of bound phenolics may become noticeable later in the digestion process, particularly in the colon, where they can be released and metabolized by the gut bacteria, even though free phenolics contribute to early antioxidant activity. To understand the variation among bound and free phenolics is crucial for evaluating the nutritional content of cereals and for making processing methods, including fermentation, that enhance the bioavailability and functional potential of the compound.

Both forms of phenolic acids exist in legumes, but the most common is the bound phenolic acid among the overall phenolic content. The outer seed coat contains the majority of the free phenolic acids, which are easily extracted and provide instant antioxidant activity. On the other hand, the majority of phenolic acids found in legumes, including ferulic, p-coumaric, caffeic, and gallic acids, are found in bound forms that are ether-linked or esterified to proteins, lignin, and cell wall polysaccharides. These bound phenolics need to be hydrolysed by enzymes or microbes in the stomach or pre-treated via techniques like fermentation, soaking, or germination in order to be released because they are not bio-accessible in their natural state (Jiang et al., 2023).

Although bound phenolics may provide delayed but long-lasting health advantages, especially in the colon, where microbial fermentation might release these chemicals, free phenolics contribute to early-phase antioxidant effects. Optimizing the nutritional and therapeutic potential of legumes, particularly in the prevention of chronic diseases and the improvement of gut health, requires an understanding of the distribution and behavior of bound vs. free phenolic acids. Phenolic acids are mostly found in fruits in their free form, while a sizable amount is also found in bound forms, especially in the skin and peel. Because free phenolic acids are easily extracted and soluble, they immediately increase the fruit’s antioxidant activity and guarantee bioavailability after intake (Gonçalves et al., 2017). These include substances that are frequently linked to the fruit’s flavour, colour, and oxidative stability, such as caffeic, gallic, and p-coumaric acids. Fruits such as berries (blueberries, strawberries, blackberries) and grapes are particularly rich in hydroxybenzoic and hydroxycinnamic acids, including gallic, caffeic, p-coumaric, and ferulic acids. Vegetables such as carrots, spinach, broccoli, tomatoes, and potatoes also contain significant levels of phenolic acids, with ferulic and caffeic acids being the most prevalent (Table 1). These natural food-derived phenolic acids contribute directly to the antioxidant capacity, flavour, and health-promoting effects of diets, and their release and bioavailability can be further enhanced through processing techniques such as fermentation, germination, or enzymatic treatment.

**TABLE 1:** Diverse dietary phenolic substances with their sources and its biological functions.

Phenolic compound	Food sources	Biological functions	References
Quercetin	Onions, apples, berries, grapes, broccoli	Antioxidant, anti-inflammatory, antihypertensive, anticancer	Aghababaei and Hadidi (2023)
Catechins (EGCG)	Green tea, apples, cocoa, berries	Antioxidant, cardioprotective, anti-obesity, neuroprotective	Cione et al. (2019)
Resveratrol	Red grapes, red wine, peanuts, berries	Anti-aging, cardioprotective, anti-inflammatory, anti-diabetic	Koushki et al. (2018)
Curcumin	Turmeric	Anti-inflammatory, antioxidant, anti-cancer, neuroprotective	Hewlings and Kalman (2017)
Chlorogenic acid	Coffee, apples, pears, potatoes	Antioxidant, anti-diabetic, lipid-lowering, hepatoprotective	Nguyen et al. (2024)
Ellagic acid	Pomegranates, berries (raspberry, strawberry)	Antioxidant, anti-cancer, antimicrobial, hepatoprotective	Evtyugin et al. (2020)
Gallic acid	Tea, grapes, berries, gallnuts	Antioxidant, anti-cancer, antimicrobial, anti-inflammatory	Kahkeshani et al. (2019)
Ferulic acid	Whole grains, oats, rice bran, corn	Antioxidant, anti-inflammatory, skin-protective, neuroprotective	M. A. Alam (2019)
Caffeic acid	Coffee, apples, berries, artichokes	Antioxidant, antimicrobial, anti-inflammatory	Birková et al. (2020)
P-coumaric acid	Tomatoes, carrots, garlic, cereals	Antioxidant, anti-inflammatory, hepatoprotective	Sova and Saso (2020)
Anthocyanins	Blueberries, blackberries, red cabbage	Antioxidant, anti-obesity, vision-improving, anti-cancer	Salehi et al. (2020)
Luteolin	Celery, green pepper, chamomile tea	Anti-inflammatory, anticancer, antioxidant, neuroprotective	Muruganathan et al. (2022)

**FIGURE 1:**
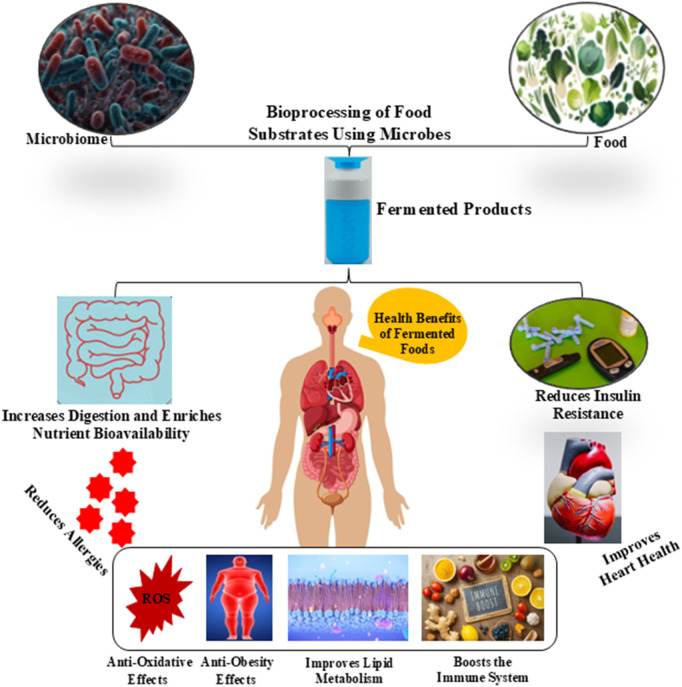
Effect of bacterial bioprocessing on the health profits of food products.

On the other hand, within the cell wall matrix, bound phenolic acids are either glycosidically or esterified to structural elements such as cellulose, hemicellulose, and pectin. These bound forms need enzymatic action or microbial fermentation in the colon to be released and activated because they are difficult to absorb in the upper gastrointestinal tract. The bound phenolic fraction, which has long-lasting antioxidant properties and prebiotic advantages, can be important for colon health even though it is usually disregarded (Siemińska-Kuczer et al., 2022). Thus, knowing the difference between free and bound phenolics in fruits is essential to appreciating their overall nutritional value and to creating food processing techniques that maximize the release and utilization of bound phenolics, particularly in fruit-based functional foods (Figure 1).

## 3 Chemical structure

According to Abbas study, phenolic compounds can be further divided into simple phenols, which have a single phenol unit, and polyphenols, which have several connected phenol units. Because of their high phenyl benzopyran content and phenillen-ene-stilbeni structure, which is shaped like this: walks of the basis of two adjacent aromatic rings (rings A and B) with the connection pyran-ring (C6-C3-C6 core structure), and because of the structural molecular between their members that occurs in quantity with a heterocyclic pyran, flavonoids are characterized as highly reactive (Abbas et al., 2017). They are bioactive due to the hydroxyl and methoxyl groups in their structure. While anthocyanins are naturally occurring pigments that are sensitive to pH and help with food preservation and coloring, flavonoids, such as quercetin, are recognized for their antioxidant stability. The recognized family of flavonoids, condensed tannins (also known as proanthocyanidins), includes polymeric phenolic compounds as well. These molecules have astringent effects but may help stabilize food systems. Many people use these subclasses of flavonoids as natural colors, stabilizers, and antioxidants. Non-flavonoid components such as phenolic acids, stilbenes, lignans, coumarins, hydrolyzable tannins, quinones, curcuminoids, and xanthones are important for food preservation and quality (Chiorcea-Paquim et al., 2020). For example, they include phenolic acids, which are found in many fruits and grains and have a C6-C1 structure, and hydrolyzable tannins, which are generated from phenolic acids and have a more complex structure (Goławska et al., 2023). They can be used for oils, beverages, and processed foods, which aligns with the sustainability trend and consumer demand for less expensive, healthier products without additives (Rana et al., 2022). Flavonol, flavone, anthocyanin, and other members of the flavonoid class are well-known for their antioxidant qualities and ability to affect color. Additionally, phenolic acids, tannins, and stilbenes are among the several other non-flavonoids that enhance the food’s durability and sensory quality. In addition to identifying socioeconomic problems, the usage of phenolic compounds and other projections towards food also serves as an enhanced bioactive compound with supplementary advantages and provides a commercial benefit (Table 1).

The food matrix is essential in influencing the release, bioaccessibility, and absorption of phenolic compounds within the human gastrointestinal tract. Phenolic compounds are frequently integrated into intricate food structures, such as dietary fibers, proteins, lipids, and cell wall polysaccharides, which may either obstruct or promote their release during the digestive process. In whole grains, legumes, and fruits, a considerable amount of phenolic acids is found in bound forms, linked to structural elements like lignin and hemicellulose, which restricts their availability for absorption in the small intestine.

The physical state of the food and the methods of food processing can significantly influence the disintegration of these matrices, thus enhancing or diminishing phenolic release. For instance, fermentation and thermal processing can break down cell walls and release bound phenolics, thereby improving their solubility and uptake in the intestines (Hassan et al., 2022). Additionally, the presence of macronutrients, such as fats, can affect the movement of phenolics through intestinal membranes by promoting micelle formation. In contrast, proteins may adhere to phenolics and minimise their absorption. Therefore, enhancing the bioavailability and functional usefulness of phenolics requires an insight into their interactions with the food matrix. In particular, while developing phenolic-rich functional foods and dietary methods aimed at averting chronic diseases.

## 4 Role as an antioxidant

**FIGURE 2:**
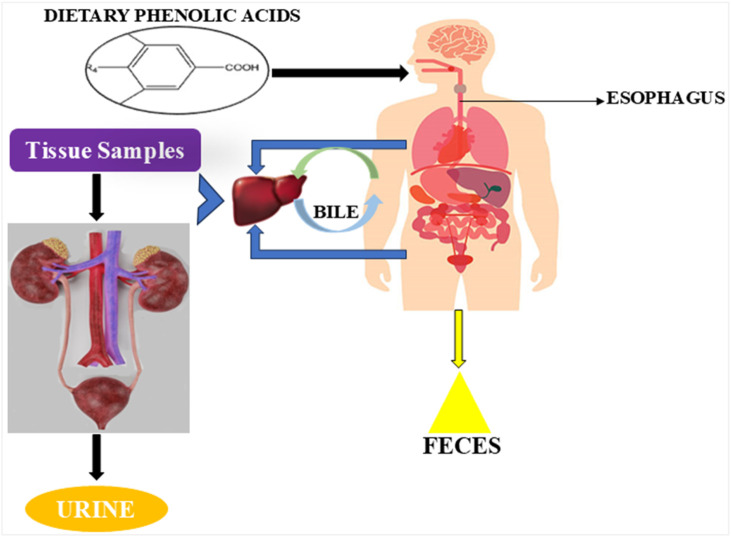
Phenolic acid metabolic pathway specifically in humans.

Flavonoids and non-flavonoids are the two primary categories of polyphenols, according to Abbas’ research. These groups encompass several subclasses, including stilbenes, phenolic acids, and tannins. These molecules’ chemical and structural makeup determines their bioactive qualities, which include antioxidant and antibacterial capabilities. Furthermore, numerous reported bioactive properties are believed to be beneficial for human health (Abbas et al., 2017). As a result, the attributes of phenolic compounds render them effective agents for the preservation and protection of plant cells against oxidative stress. While tannins and stilbenes play significant roles in defending against microbes and extending the shelf life of plant-derived products, phenolic acids offer protective benefits against pathogens and free radicals. Figure 2 exemplifies the general metabolic pathways of dietary phenolic acids. The excretory system eliminates the waste products from the metabolism of phenolic acids, mostly in urine and feces (Teshome et al., 2022).

## 5 Bacterial biotransformation

### 5.1 Microbial-based biotransformation

Due to benefits such as mild reaction conditions and excellent substrate specificity, biotransformation technologies have emerged as a crucial method for the targeted structural alteration of phenolic acids and the creation of high-value derivatives. The bulk of phenolic acid synthesis techniques currently in use are chemical, microbiological, and enzymatic; nevertheless, microbial and enzymatic methods have garnered more attention in research due to concerns about food safety (Tembeni et al., 2024; Cao et al., 2025). Phenolic acids obtained from plant biomass and agro-industrial waste can undergo various microbial and enzymatic transformations, resulting in products that are both nutritionally and industrially valuable. Major pathways include decarboxylation, which produces hydroxy-styrene derivatives, amination, yielding compounds like vanillylamine, and ferulic acid degradation, resulting in vanillin and cis,cis-muconic acid. Furthermore, esterification, hydroxylation, halogenation, and acid/aldehyde reduction lead to derivatives such as phenolic esters, 3-hydroxytyrosol, and halide conjugates. Microbial degradation of aromatics and the design of synthetic pathways also allow for the conversion of phenolic acids into organic acids (succinic and lactic) and biofuels (Lu et al., 2025; Tinikul et al., 2018). Bacterial techniques primarily utilise the metabolic capacities of microorganisms to biotransform phenolic acids into useful chemicals. *Aspergillus niger*, *Lactobacillus* species, and *Saccharomyces* yeasts are among the several microbiological taxa that have the ability to biotransform into phenolic acids. For example, following the fermentation of wheatgrass juice, *Saccharomyces cerevisiae* and *Pediococcus acidilactici* BD16 facilitated the biotransformation of phenolic acid, greatly improving the nutritional and functional properties of the juice (Akpabli-Tsigbe et al., 2023).

Solid-state fermentation (SSF) with *Lactobacillus casei* and *L. helveticus* offers an efficient strategy to release bound phenolic acids, particularly chlorogenic acid, from soybean matrices. The optimized SSF not only facilitated the cleavage of protein/carbohydrate–phenolic linkages but also enhanced antioxidant bioactivity, with structural changes validated by SEM, AFM, and FTIR analyses. Akpabli-Tsigbe et al. (2023) further demonstrated that such mixed-culture fermentation effectively disrupted macromolecular bonds in soybean cell walls, underscoring the potential of lactic acid bacteria-driven biotransformation in upgrading low-cost substrates into value-added phenolic compounds. In another study, solid-state fermentation of *Citrus reticulata* peel with *A. niger* CGMCC 3.6189 enhanced antioxidant activity by increasing phenolic and flavonoid contents. Microbial biotransformation converted native flavonoids (hesperidin, nobiletin, and tangeretin) into more bioactive phenolic acids, such as p-coumaric and ferulic acids, while SEM and FTIR confirmed the structural modifications. This demonstrates the role of *A. niger* in upgrading citrus byproducts into antioxidant-rich functional ingredients (Mamy et al., 2024).

In wheatgrass juice, Kaur et al. (2023) used two-stage fermentation with *S. cerevisiae* and recombinant *P. acidilactici* BD16 (alaD+), which showed enhanced anthocyanins, phenols, and β-carotenes compared to unfermented juice. Yeast and lactic acid bacteria mediated diverse phenolic biotransformations, including conversion of hydroxycinnamic and hydroxybenzoic acids, flavonoid glycosylation, and carotenoid synthesis, while also supporting lignin glycosylation and derivatisation of benzoic and quinic acids. Similarly, Huang et al. (2022) improved the mixed-strain fermentation (*Bacillus subtilis*, Pediococcus acidilactici, and *Candida tropicalis*) of rapeseed meal, where a diverse range of microbes helped release bioactive polypeptides. Meanwhile, Zhou et al. (2020) successfully engineered *Pseudomonas putida* KTc9n20 to accomplish scalable ferulic acid valorisation after conducting extensive research on the conversion of ferulic acid to polyhydroxyalkanoates (PHA). By changing free ferulic acid to 4-vinyl guaiacol and acetovanillone, *Streptomyces tunisiensis* DSM42037 notably exhibited remarkable catalytic efficiency, with molar biotransformation resulting in 97% and 83%, respectively (Zhou et al., 2020). Highly effective biotransformation of phenolic acids is accomplished by microbial methods; certain strains allow for quick conversion in brief periods. Furthermore, microbial processes offer substantial sustainability and environmental benefits. They can change the structure and function of different phenolic groups by enhancing their biological activity and accessibility, and also making them more physiologically active. They can also prolong food goods’ shelf life and improve their sensory quality (Slama et al., 2021). These examples emphasize the broader applicability of microbial biocatalysis, from nutraceutical and functional food enhancement to sustainable biopolymer production, offering both health and environmental benefits.

### 5.2 Enzymes in the biotransformation process

**FIGURE 3:**
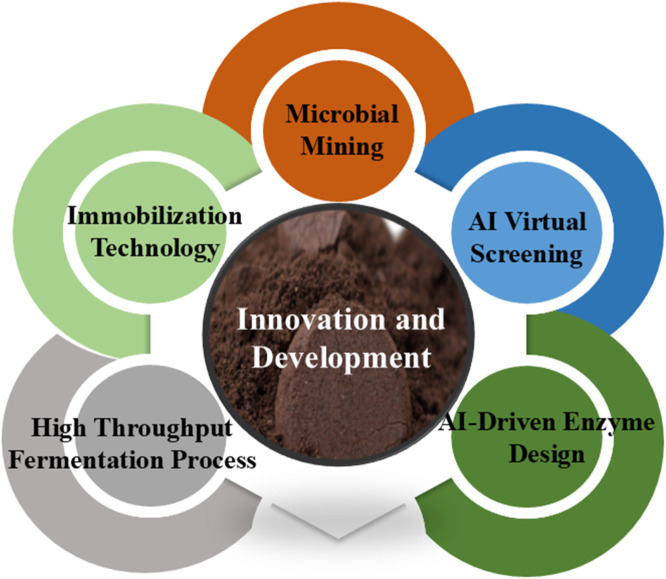
Combined development and innovation of essential enzymes for the biotransformation of phenolic acids.

Major catalytic enzymes involved in the biotransformation routes of phenolic acids include phenolic acid decarboxylase, phenolic acid esterase, phenolic acid reductase, and β-glucosidase. Through a variety of catalytic processes, these enzymes work together to assist successive phases of reactions, forming the enzymatic framework responsible for phenolic acid biotransformation (Ji et al., 2024). By using particular enzymes as biocatalysts, the enzymatic method is a biotransformation technology that improves the biotransformation of phenolic acids through enzyme-catalyzed processes. Enzymatic techniques provide higher selectivity and specificity than other biotransformation techniques, allowing for accurate catalysis of specific processes. This method minimizes energy consumption while successfully reducing the possible deterioration of substrates and goods brought on by extreme heat or pressure. Mild working conditions, usually kept at room temperature and pressure, are its defining feature. However, there are a number of technical difficulties with enzymatic methods (Ai et al., 2022). Enzyme extraction and purification are costly and intricate processes, and their inherent instability further restricts their use on an industrial scale (Figure 3).

To overcome these limitations and enhance enzymatic stability and efficiency in phenolic acid biotransformation, researchers have developed state-of-the-art technical solutions. In immobilized enzyme technology, for instance, enzyme fixation on particular matrices significantly improves heat stability. Furthermore, novel strategies in synthetic biology and metabolic engineering optimize microbial systems for enhanced catalytic activity and the synthesis of critical enzymes using genetic modification techniques. Because of its incredible potential for application in the food, pharmaceutical, and cosmetic industries, phenolic acid biological transformation technology has garnered a lot of attention (Wang et al., 2023).

Using biotechnological methods such as enzymatic catalysis and microbial fermentation, phenolic acids are precisely bio-transformed into bioactive derivatives with enhanced antioxidant activity. This process provides a scientific foundation for the development of functional foods and nutraceuticals. In contrast to enzymatic methods, the elucidation of microbial metabolic pathways for phenolic acids has enabled the identification and functional characterisation of significant enzymes. By removing necessary enzymes from microorganisms and improving their catalytic activity, researchers avoid the challenges of microbial cultivation and enable direct and efficient phenolic acid biotransformation (Wang et al., 2024). Furthermore, technical advancements have sped up the development of enzymatic strategies, as demonstrated by enzyme engineering and immobilization techniques that significantly improve catalytic efficiency and operational stability.

#### 5.2.1 Phenolic acid decarboxylase

##### 5.2.1.1 Structure of phenolic acid decarboxylase

Phenolic acid decarboxylase (PAD) is a significant category of enzymes that leads to the decarboxylation of phenolic acid derivatives. By selectively eliminating the carboxyl group (-COOH), this enzyme delivers volatile 4-vinylphenolic derivatives, like 4-vinylguaiacol and 4-vinylphenol. PAD is broadly distributed in plants and bacterial schemes are important for food fermentation and natural product synthesis (Deng et al., 2024). Because molecule conformation as well as arrangement of key domains directly affect substrate identification, reaction efficiency, and environmental adaptation, PAD’s structural design is precisely matched to its catalytic functions. PAD is a member of the superfamily of α/β-hydrolases and is usually exist as homo-dimers or -tetramers. Its fundamental structure, which consists of parallel or mixed β-sheets surrounded by α-helices, has a distinctive “α/β-barrel” folding pattern. A stable hydrophobic microenvironment is produced by this arrangement, which is necessary for substrate binding and catalytic activity (Bierbaumer et al., 2023).

##### 5.2.1.2 Mechanisms of phenolic acid decarboxylase

With the previous research mostly attributing the decarboxylation process to synergistic interactions among conserved amino acid residues inside the active site, our understanding of the PAD catalytic mechanism is still restricted. There are four major stages that the reaction goes through: First, a glutamate residue mediates the protonation of the substrate’s phenolic hydroxyl group (Sheng et al., 2015). A nucleophilic center is then formed at the carbon next to the carboxyl group as a result of electronic rearrangement. At the same time, asparagine (Asn) acts as a proton donor to help create a quinoid transition-state intermediate by stabilizing a water molecule through hydrogen-bonding networks. Ultimately, volatile 4-vinylphenolic derivatives are the reaction result, and CO_2_ is produced. The study demonstrates that the PAD catalytic core is dominated by acid–base catalysis, with a hydrophobic cavity in the active site that is essential for substrate binding and reaction efficiency (G. Tian and Liu, 2017). The functional distinction between the enzyme’s terminal extensions is further demonstrated by structural analyses: the N-terminal extension seems to maximize substrate binding in alkaline environments through better cavity interactions and structural stabilization, whereas the C-terminal extension improves acid tolerance. Furthermore, a study discovered a noteworthy regulatory effect of reducing agents, such as homocysteine, cysteine, 2-mercaptoethanol, and dithiothreitol (DTT), which markedly increased the ferulic acid decarboxylation activity in both native and recombinant CgPAD. Interestingly, these substances had no discernible effect on the decarboxylation activity of p-coumaric acid, indicating that redox-sensitive molecular interactions modulate enzyme function substrate-specifically (Huang et al., 2012).

#### 5.2.2 Phenolic acid esterase

##### 5.2.2.1 Structure of phenolic acid esterase

**FIGURE 4:**
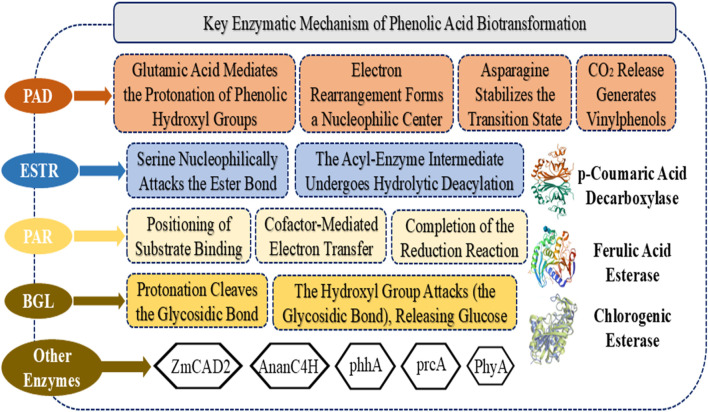
Key enzymatic mode of action of phenolic acid biotransformation.

Phenolic Acid Esterase’s Structure As an essential member of the carboxylesterase family, phenolic acid esterase is a key catalyst in biological metabolic processes. By selectively hydrolyzing ester bonds in phenolic acid derivatives, this enzyme contributes to a variety of biotransformation pathways. ESTR is widely found in animals, microbes, and plants and exhibits both substantial biological significance and potential for industrial use (Li et al., 2024). The conserved Ser-His-Asp catalytic triad forms the active centre of ESTR, which structurally displays the distinctive α/β hydrolase fold structure. Histidine (His) serves as a general base to activate the serine hydroxyl group and facilitate proton transfer, whereas the serine residue (Ser) acts as a nucleophile to directly target the carbonyl carbon of the ester bond (Rauwerdink and Kazlauskas, 2015). By stabilizing histidine’s protonation state, aspartate (Asp) preserves the catalytic microenvironment (Silveira et al., 2021; Xu et al., 2020). Because of its unique structural makeup, ESTR has exceptional hydrolytic efficiency, which permits it to exhibit specialized activity toward hydroxycinnamate esters and aids in vital metabolic processes in biological systems (Figure 4).

##### 5.2.2.2 Mechanisms of phenolic acid esterase

ESTR uses the fundamental Ser-His-Asp catalytic triad as its catalytic mechanism to carry out a two-step nucleophilic activity. A tetrahedral transition state intermediate is first formed when the serine residue in the active site starts a nucleophilic attack on the carbonyl carbon of the ester bond. In order to achieve effective substrate conversion, the acyl-enzyme intermediate then goes through hydrolytic deacylation. The wolfberry fermentation system serves as an example of this catalytic process (Liu et al., 2024). Chlorogenic acid is particularly hydrolyzed into quinic acid and caffeic acid by the extremely effective ESTR system of *Lactobacillus plantarum* NCU137. In addition to improving the fermented goods’ flavor character, this biotransformation process produces phenolic acid derivatives with strong antioxidant properties. These results offer important new information for the creation of functional foods (Lin and Gänzle, 2014).

#### 5.2.3 β-glucosidase

##### 5.2.3.1 Structure of β-glucosidase

As a member of the hydrolase superfamily’s glycosidase family, β-glucosidase catalyzes the hydrolysis of glycosidic bonds that connect terminal non-reducing sugar residues to aromatic/alkyl aglycones or oligosaccharides (Han et al., 2022). As is typical of glycoside hydrolase families like GH1 and GH3, β-glucosidases structurally adopt a conserved (β/α) 8 barrel catalytic domain. Two essential acidic amino acid residues, such as glutamic acid (Glu) and aspartic acid (Asp), are present in the active site and serve as general bases and acids, respectively (Liang et al., 2022; Yao et al., 2024). To guarantee the stereospecific identification of the β-configuration, substrate binding entails hydrogen bonding and hydrophobic interactions between the conserved residues (such as tyrosine and tryptophan) in the active site and the glucosyl moiety of the β-glycosidic bond. At the same time, a hydrophobic pocket stabilizes the aglycone moiety (Zhong et al., 2021).

##### 5.2.3.2 Mechanisms of β-glucosidase

Two consecutive steps make up the catalytic mechanism: Protonation is first caused by the general acid residue, giving the glycosidic oxygen a proton (H+). The aglycone is released as a result of the weakening of the glycosidic link, which causes it to cleave and generate a glycosyl-enzyme covalent intermediate. A nucleophilic hydroxide ion (OH−) is then produced when the general base residue abstracts a proton from a water molecule to activate it. By attacking the glycosyl-enzyme intermediate’s anomeric carbon, this ion releases β-D-glucose and returns the enzyme to its active form. β-glucosidases from various origins differ in their cofactor needs or catalytic residues. For example, Trametes trogii S0301's GH3 family member TtBgl3 has remarkable phenolic compound deglycosylation activity, making it a prospective green biocatalyst for the synthesis of bioactive glycosides for use in biotechnological and medicinal applications (Qu et al., 2022).

## 6 Case studies of phenolic acids from fermented food-based systems

Fermentation has become a significant method for improving the profile of bioactive compounds, especially phenolic acids, in plant-based foods, including maize, quinoa, soy, and various fruits. In the context of fermented maize, research indicates that the use of lactic acid bacteria (LAB) and bifidobacteria leads to a notable increase in free phenolic acids such as caffeic, syringic, vanillic, and protocatechuic acids. Conversely, the levels of compounds like ferulic and p-coumaric acids appear to decline, which implies microbial metabolism and transformation. This fermentation process not only enhances the antioxidant capacity of maize but also produces a gluten- and lactose-free probiotic snack that maintains stable microbial viability and is well-received by consumers (Adebo et al., 2022).

In fermentation systems based on quinoa, both fungal and bacterial processes significantly modify the phenolic profile. Fungal fermentation involving *Rhizopus oligosporus, Rhizopus microsporus*, and *Saccharomyces cerevisiae* facilitates the enzymatic liberation of bound hydroxybenzoic and hydroxycinnamic acids. For instance, the conversion of ferulic acid into vanillin and the elevation of gallic, protocatechuic, and vanillic acids have been documented. These alterations are particularly evident in colored quinoa varieties, where the flavonoid content, including catechin, kaempferol, and quercetin, initially increases before undergoing degradation over time (Melini and Melini, 2021). LAB fermentation with Lactiplantibacillus plantarum 299v® significantly boosts total phenolic content (TPC), rising from 4.7 to 7.8 mg GAE/100g in white quinoa and from 5.0 to 8.1 mg GAE/100g in red quinoa. Remarkably, there was an 18-fold increase in epicatechin in white quinoa, while red quinoa exhibited a substantial increase in catechin (∼59 μg/g). These biochemical modifications are associated with a notable enhancement in antioxidant activity, as assessed by DPPH and FRAP assays. When quinoa undergoes both cooking and fermentation with strains such as *Lb. paracasei* and *Pediococcus pentosaceus*, the release of phenolics and antioxidant levels further rise, indicating a synergistic effect of thermal and microbial processing (Nardini, 2023).

In soy-based systems, fermentation primarily improves the bioavailability of isoflavones by transforming their glycoside forms into aglycones through the action of microbial β-glucosidases. Lactic acid bacteria (LAB) like *Lb. acidophilus* and *Lb. casei*, along with fungal strains such as Aspergillus and Rhizopus, are particularly adept at facilitating this conversion (Chu et al., 2024). These aglycones demonstrate significantly enhanced antioxidant activity and are absorbed more efficiently in the human gastrointestinal tract. Research involving animals has further shown that fermented soymilk produced with *Lb. fermentum* CQPC04 improves markers of oxidative stress and supports gut health, thereby reinforcing the functional advantages of phenolic enhancement through fermentation.

In fruit-based systems, solid-state fermentation (SSF) of mango seed by *Aspergillus niger* GH1 has demonstrated a remarkable increase in total phenolic content (TPC), rising from approximately 984 mg to over 3288 mg GAE/100g within 20 h. This significant enhancement is attributed to the enzymatic actions of the fungus, which liberate bound phenolics from the fruit matrix, thereby augmenting antioxidant potential (Lei et al., 2024). These results highlight the effectiveness of fungal SSF in converting low-value by-products into high-value functional ingredients. In these systems, the common mechanisms that facilitate the enhancement of phenolic acids include the enzymatic degradation of plant cell walls, hydrolysis of glycosidic bonds, and microbial transformation of precursor compounds. The specific results, such as the increase or decrease of particular phenolics, are largely influenced by the type of microorganism used, the duration of fermentation, and the intrinsic composition of the food matrix (Khan et al., 2024). Typically, there is a significant improvement in antioxidant activity, and health-promoting compounds become more bioavailable. Nevertheless, additional research, especially human clinical trials, is essential to translate these biochemical advancements into dietary and therapeutic guidelines effectively. Furthermore, the optimization of fermentation conditions and the selection of strains remain vital areas for the progression of the functional food sector.

## 7 Quantitative change in phenolic acid profiles

Significant alterations in the quantities of specific phenolic compounds in response to environmental factors or elicitor applications are shown by quantitative variations in phenolic acid profiles. An important illustration is the rise in caffeic acid levels, which have been observed to increase by 21%–151% in situations involving stress or following the administration of elicitors like methyl jasmonate and salicylic acid (Valanciene et al., 2020). This rapid rise indicates that the phenylpropanoid pathway, which promotes the manufacture of caffeic acid, a substance known for its potent antibacterial and antioxidant qualities, has been activated. These metabolic modifications enhance the plant’s nutritional and therapeutic qualities and are essential to its defensive mode of action. For instance, the focused interventions can change phenolic acids to increase the biologically active production. Goswami’s report showed, caffeic acid contents in *Ocimum sanctum* improved by 21%–151% after elicitor treatment (Goswami et al., 2020; Li et al., 2023). Significant variations in gallic acid accumulation are signified by quantitative alterations in gallic acid profiles that are recurrently brought on by biotic or abiotic stressors followed by the usage of elicitors (Valério et al., 2021; Zhao et al., 2021). Known for its strong antibacterial and antioxidant properties, gallic acid is produced via the shikimate pathway and is essential to plant defense systems. The concentration of gallic acid can rise dramatically after being treated with elicitors such as methyl jasmonate or salicylic acid. Gallic acid levels in Ocimum sanctum, for instance, increased dramatically after elicitor treatment, rising by 18%–139%. Together with a 21%–151% increase in caffeic acid, this rise indicates that secondary metabolic pathways targeted at enhancing the plant’s resistance and medicinal qualities have been activated (Jain et al., 2024). The potential of elicitor-based techniques to increase the synthesis of bioactive compounds in medicinal plants is highlighted by these quantitative changes (Table 2).

**TABLE 2:** Phenolic acid biotransformation by microbes during fermentation.

Name of phenolic acid	Food specimen	Strains utilized	Enzymes involved	Results of biotransformation	References
Ferulic acid	Wheat, Maize	*Lactobacillus* spp.	Feroloyl esterase	Release from bound form	Deng et al. (2019)
Ferulic acid	Wheat bran	*Lactobacillus plantarum*	Feruloyl esterase	65.3% rise in free ferulic acid	Zhang et al. (2022b)
p-Coumaric acid	Fermented vegetables	*Lactobacillus brevis*	Phenolic acid decarboxylase	Conversion to 4-vinylphenol and increased free p-coumaric acid	Filannino et al. (2015)
Caffeic acid	Coffee pulp	*Saccharomyces cerevisiae*	Caffeate reductase, methyltransferase	Bioconversion to ferulic and dihydrocaffeic acid	Chiang et al. (2014)
Gallic acid	Pomegranate peel	*Aspergillus niger*	Tannase, gallate decarboxylase	70% increase in gallic acid via hydrolysis of gallotannins	Mahendran et al. (2024)
Chlorogenic acid	Green coffee beans	*Lactobacillus rhamnosus*	Esterase	Hydrolysis to caffeic acid, reduction of chlorogenic acid	Hur et al. (2018)

## 8 Structure-activity relationships and antioxidant potential

A phenolic molecule can demonstrate antioxidant activity through a hydrogen atom transfer (HAT) mechanism, sequential proton loss electron transfer, single-electron transfer coupled with proton transfer (SET-PT), or by chelation with transition metals (TMC). Transfer of hydrogen atoms Free radicals and antioxidants with H-atoms, shown by AH, interact in HAT. While the antioxidant is transformed into an antioxidant free radical (A*), the free radical is stabilized to produce a neutral species. Phenolic antioxidant (AH) can supply an H-atom to the free radical substrate, resulting in the production of an antioxidant free radical (A*) and non-radical substrate (RH, ROH, or ROOH) species (Zhang and Tsao, 2016; Lee et al., 2020). The reduction potential explains why an antioxidant donates a hydrogen atom. According to Shahidi and Ambigaipalan (2015), “Unless the reaction is kinetically unfeasible, any compound that has a reduction potential lower than the reduction potential of a free radical (or oxidized species) is capable of donating its hydrogen atom to that of the free radical.” The structure of phenolic compounds, particularly the benzene ring and the quantity and location of the OH group, determines their capacity or potency to have an antioxidant effect (Shahidi and Ambigaipalan, 2015). Antioxidant molecules stabilize when they react with free radicals because of the benzene ring. Gallic acid is a phenolic acid that has one carboxylic acid group and three hydroxyls. However, by creating gallic acid-free radicals, the hydroxyl group is in charge of the antioxidant activity. The resonance actions of the aromatic ring stabilize the radical. Flavonols with five hydroxyl groups, like quercetin, have a relatively higher antioxidant capacity than those without, as they can neutralize free radicals (Shahidi and Ambigaipalan, 2015).

If the COOH group has more space, such as between the CH2 group and the benzene ring, the antioxidant activity of phenolic acid is increased. The longer space impacts the COOH group’s ability to extract electrons. For instance, compared to benzoic acid or its derivatives, cinnamic acid or its phenolic derivatives exhibit greater antioxidant activity. Likewise, the location of certain groups, like hydroxyl groups, on the benzene ring influences its antioxidant capacity. For instance, hydroxyl groups at the ortho and/or para positions exhibit higher levels of antioxidant activity than unsubstituted phenol and the meta position. increases the antioxidant’s activity when an ethyl or n-butyl group is substituted for a methyl group at the para-position. However, the presence of an alkyl chain or branched alkyl groups on the para position resulted in a decrease in the antioxidant activity. As demonstrated in butylated hydroxytoluene (BHT), the stability of radicals of an aromatic ring is further enhanced when bulky groups are included at positions two and six. The large groups lower the rate of free radical propagation reactions by adding to the steric barrier in the radical area. The antioxidant potential is also influenced by the quantity of hydroxyl groups on the benzene ring. For instance, it has been demonstrated that flavonoids exhibit greater antioxidant activity than phenolic acids (Rouaiguia-Bouakkaz and Benayahoum, 2015). It is important to note that one of the most important factors in determining the function of an antioxidant is the bond dissociation energy (BDE) of the H-atom connected to the oxygen atom of phenolic.

### 8.1 Single electron transfer

While ArOH (the cation radical) is a less reactive radical species and hence stable, the R (anion) generated in the single electron transfer (SET) process is an energetically stable species with an even number of electrons. An aromatic ring structure with the potential to be dispersed across the entire molecule is the odd number of electrons created by the reactions with the radical in ArOH+*. The most crucial restriction for assessing scavenging activity in the SET mechanism is the ionization potentials (IPs) of various atoms. A lower IP value will result in easier electron-abstraction and the ensuing reaction with a radical (Leopoldini et al., 2011). A high level of antioxidant activity was the outcome. Chelation of transition metals. One of the antioxidant functions of phenolic compounds is transition metal chelation (TMC). Polyphenols bind transition metals to generate stable compounds, according to experiments. Such processes can be catalyzed by transition metals like cobalt (Co.), manganese (Mg), and copper (Cu). However, there are some restrictions to chelation, like the metal ions’ inability to bind to proteins or other chelator molecules. Food-based phenolic compounds are excellent metal chelators (Apak et al., 2016).

However, depending on their structural features, the reducing power and chelating potential with metals may vary. For instance, the catechol moiety (ring B), the 2,3-double-bonded and conjugated 4-carboxylic group in the C-ring, and the 3 and 5-hydroxyl moieties are necessary for flavonoids to have stronger metal chelating and radical scavenging capabilities (Mathew et al., 2015). Compared to Fe2+, the polyphenol ligands significantly stabilized Fe3+. The Fe2+ complexes of gallate and catecholate, for instance, rapidly oxidized to form Fe3+-polyphenol complexes when exposed to oxygen. Auto-oxidation is a typical term for this process. In the presence of oxygen, Fe2+ typically oxidizes slowly. However, as soon as polyphenol ligands attach to Fe2+, the metal’s reduction potential is dropped, and the rate of Fe oxidation is accelerated. Gallate complexes oxidize at a quicker pace than catecholate complexes, indicating that the oxidation rate of iron varies among polyphenol complexes. The increased stability of Fe3+ interactions with the hard oxygen ligands of the phenol moieties facilitates the oxidation of iron following attachment to phenol ligands. Another factor is the oxygen ligands’ potent electron-giving properties, which help to stabilize the Fe3+ state (Espina et al., 2022).

The antioxidant properties of phenolic acids are greatly affected by their patterns of hydroxylation and methoxylation. Hydroxyl groups are essential for neutralizing free radicals through the donation of hydrogen atoms, with their position and quantity directly influencing the radical-scavenging efficiency of the compound. Phenolic acids that possess ortho-dihydroxyl groups, such as caffeic acid, demonstrate improved antioxidant activity due to the stabilization of semiquinone radicals through intramolecular hydrogen bonding and resonance. Conversely, hydroxyl groups located in para or meta positions contribute less to the stabilization of radicals (Mathew et al., 2015). In contrast, methoxylation generally diminishes antioxidant activity by limiting the availability of free hydroxyl groups for hydrogen donation. For instance, ferulic acid, which includes a methoxy group, exhibits lower radical scavenging activity compared to caffeic acid, despite their structural similarities. Nevertheless, methoxy groups can enhance lipophilicity, thereby improving membrane permeability and potentially increasing biological efficacy in lipid environments. In conclusion, an increased level of hydroxylation, particularly in ortho positions, enhances antioxidant capacity, whereas methoxylation frequently has a modulating or sometimes antagonistic influence, contingent upon the position and quantity of substituents (Chen et al., 2020; Fitsum et al., 2025). When found in fermented food matrices, phenolic acids often work in conjunction with peptides and short-chain fatty acids (SCFA) to enhance the bioactivity and health benefits of these compounds. Bioactive peptides and SCFAs, including acetate, propionate, and butyrate, can be released by microbial metabolism during fermentation (Cuciniello et al., 2023; Peng et al., 2025).

## 9 Molecular mechanisms of action

### 9.1 Activation of Nrf2–ARE pathway

Several phenolic acids, including ferulic acid, caffeic acid, sinapic acid, and protocatechuic acid, have been shown to activate the Nrf2–ARE (antioxidant response element) signaling pathway, a central regulator of cellular defense against oxidative stress. Ferulic acid induces Nrf2 nuclear translocation and upregulates heme oxygenase-1 (HO-1) expression in endothelial and neuronal cells, thereby reducing oxidative damage (Kumar and Pruthi, 2014; Chen et al., 2022). Similarly, caffeic acid and its derivative caffeic acid phenethyl ester (CAPE) activate Nrf2-dependent HO-1 and NAD(P)H quinone dehydrogenase 1 (NQO1) expression, conferring cytoprotection (Yang et al., 2019). Sinapic acid has also been reported to enhance antioxidant enzyme activities through the pathway of nuclear factor-erythroid 2-related factor-2 (Nrf2)/heme oxygenase 1 (HO-1) signalling and through the activation antioxidant enzymes that helps in the alleviation of different toxicity conditions including osteoarthritis (Li et al., 2019), Cadmium-Induced Hepatotoxicity (Farahat et al., 2025; Ahmad et al., 2021), 5-fluorouracil-induced nephrotoxicity (Ansari et al., 2023), Doxorubicin-Induced Cardiotoxicity (Tungalag et al., 2025), and bleomycin-induced lung fibrosis (Raish et al., 2018). While protocatechuic acid increases glutathione synthesis and reduces lipid peroxidation via Nrf2-mediated pathways (Varì et al., 2011). These findings highlight the crucial role of phenolic acids in modulating redox balance through the Nrf2/HO-1 and Nrf2-ARE signalling pathways. These pathways play a vital role in adapting to oxidative stress caused by pro-oxidants and electrophiles by up-regulating phase II detoxifying enzymes.

Through non-covalent interactions, like hydrogen bonds and hydrophobic forces, peptides may enhance the solubility and stability of phenolic acids. It also enhances their capacity to scavenge radicals and enhance bioavailability (Peng et al., 2025). In a similar way, SCFAs can impact the gut environment, enhancing phenolic acid absorption and metabolic pathways, while phenolic substances may prevent SCFAs from oxidative deterioration. Yogurt, kimchi, and sourdough, where the concurrent existence of bacterial metabolites and changed phenolic acids causes enhanced physiological reactions, these synergistic interactions are more noticeable in the fermentation process (Muntaha et al., 2025). Altogether, these actions enhance gut health, regulate immunological responses, and perhaps offer defense against metabolic illnesses (Sahoo et al., 2023).

### 9.2 Suppression of NF-κB and MAPK signalling

The signalling pathways for MAPK (mitogen-activated protein kinase) and NF-κB (nuclear factor kappa-light-chain-enhancer of activated B cells) are essential modulators of cellular stress, inflammation, and immunological response. Chronic inflammation and several illnesses, including cancer, neurodegeneration, and metabolic disorders, are linked to their over activation. Therefore, preventing excessive inflammatory reactions requires blocking these pathways. In the cytoplasm, where it is bound by IκB proteins, NF-κB is normally dormant. IκB is phosphorylated and degraded in response to pro-inflammatory stimuli (such as LPS and cytokines), which permits NF-κB to go into the nucleus and stimulate the production of pro-inflammatory genes, such as TNF-α, IL-6, and COX-2. Also, kinases like ERK, JNK, and p38 are part of the MAPK pathway, which activates transcription factors like AP-1 to further promote inflammation and cell division (Anilkumar and Wright-Jin, 2024). Research shows that ferulic acid helps reduce inflammation and insulin resistance by controlling the JNK/ERK and NF-κB pathways in 3T3-L1 adipocytes treated with TNF-α, and also through the NF-κB-MAPK pathway in bovine endometrial epithelial cells, when exposed to LPS-induced inflammation (Park et al., 2024; Yin et al., 2019). Caffeic acid regulates the activation of NF-κB by LPS through the NIK/IKK and c-Src/ERK signaling pathways (Kim et al., 2014). By reducing upstream activators, preventing the nuclear translocation of transcription factors, or blocking kinase activity, natural substances, including flavonoids, phenolic acids, and other phytochemicals, can impede various signalling pathways. These bioactive chemicals have anti-inflammatory, antioxidant, and anti-cancer activities by targeting the NF-κB and MAPK pathways, providing therapeutic potential for the management of illnesses connected to inflammation (Saleh et al., 2021).

### 9.3 Variation of gut barrier integrity and redox-sensitive gene expression

The gut barrier serves as a selective physical and immunological barrier that permits the absorption of nutrients while shielding the host from dangerous microorganisms. Tight junction (TJ) proteins, such as occludin, claudins, and zonula occludens (ZO-1), maintain its integrity. Increased intestinal permeability, sometimes referred to as “leaky gut,” can result from oxidative stress or inflammation that compromises this barrier. This condition is associated with a number of gastrointestinal and systemic conditions. Through a variety of methods, phenolic acids and other bioactive substances can strengthen the intestinal barrier (Saleh et al., 2021). By blocking pro-inflammatory mediators like TNF-α and IL-1β and activating signaling pathways like AMPK, they increase the expression of tight junction proteins. Additionally, they boost the expression of redox-sensitive genes controlled by the Nrf2-ARE pathway, which raises the synthesis of antioxidant enzymes like catalase (CAT), glutathione peroxidase (GPx), and superoxide dismutase (SOD) (Table 3). By lowering intracellular ROS levels, these enzymes help shield intestinal epithelial cells from oxidative injury. Furthermore, bioactive substances influence metabolites produced by microorganisms (such as SCFAs), which can activate GPR43/109A receptors, promoting anti-inflammatory reactions and supporting the preservation of epithelial barrier function (Guo et al., 2024). To sum up, these activities are critical for preventing intestinal dysfunctions and chronic diseases because they preserve gut integrity, lower inflammation, and maintain redox equilibrium.

**TABLE 3:** Mode of actions and health impacts of phenolic acids from fermented foods.

Name of the phenolic acid	Mechanism	Target pathway	Health benefit	References
Ferulic acid	Lipid peroxidase inhibition	Nrf2-ARE, MAPK	Cardiovascular protection	Li et al. (2025)
Caffeic acid	Inhibits NF-κB activation, reduces ROS	Nrf2–ARE, Gut barrier signaling	Antioxidant, gut barrier protection	Zhu et al. (2025)
p-Coumaric acid	Modulates microbiota, promotes SCFA production	NF-κB, MAPK	Anti-inflammatory, oxidative stress reduction	Akanyibah et al. (2025)
Gallic acid	Suppresses pro-inflammatory cytokines, enhances GSH levels	GPR41/GPR43, Microbial fermentation	Improved gut health, immune modulation	Kim et al. (2006)
Sinapic acid	Enhances mitochondrial function, regulates TLR4 signaling	NF-κB, Redox-sensitive genes	Anti-inflammatory, antioxidant	Farahat et al. (2025)
Chlorogenic acid	Modulates gut microbiota, inhibits LPS-induced inflammation	TLR4, AMPK	Neuroprotection, anti-inflammatory	Gu et al. (2023)
Syringic acid	Scavenges ROS, inhibits COX-2 and iNOS expression	TLR4/NF-κB, Microbiota modulation	Gut integrity, metabolic improvement	Krayem et al. (2025)
		MAPK, COX-2/NOS pathways	Anti-inflammatory, hepatoprotective	Park et al. (2024)

### 9.4 Connections with SCFA and gut microbiota

Substantial interactions exist between phenolic acids and the gut microbiota, influencing the metabolic activities of microbes, particularly the formation of SCFAs, as well as their composition. Many phenolic substances enter the colon unmetabolized after consumption, where microorganisms transform them into bioactive metabolites. These compounds can restrict hazardous strains of bacteria while selectively promoting the growth of beneficial ones like *Lactobacillus, Bifidobacterium*, and *Faecalibacterium prausnitzii.* By improving the fermentation of dietary fibers, this microbiome modification raises the synthesis of SCFAs such as butyrate, propionate, and acetate. SCFAs support the integrity of the gut barrier, control immunological responses, provide vital energy for colonocytes, and activate G-protein-coupled receptors (GPR41, GPR43) to influence inflammation and host metabolism. Additionally, butyrate acts as a histone deacetylase (HDAC) inhibitor, influencing gene expression related to oxidative stress and inflammation. This complex interplay between phenolic acids, gut microbes, and SCFA production is crucial for maintaining both intestinal and systemic health by promoting anti-inflammatory, antioxidant, and gut-protective effects (O'Riordan et al., 2022).

## 10 Functional health implications

### 10.1 Anti-inflammatory and immunomodulatory impacts

Through a variety of molecular pathways, phenolic acids significantly influence immunomodulatory and anti-inflammatory activities. These bioactive compounds reduce the expression of cytokines, including TNF-α, IL-1β, and IL-6 by blocking important pro-inflammatory signalling pathways like NF-κB, MAPK, and JAK/STAT. They also reduce prostaglandins and nitric oxide, which are involved in inflammatory reactions, by modifying the activity of cyclooxygenase (COX-2) and inducible nitric oxide synthase (iNOS). Furthermore, through controlling macrophage polarization, boosting T-regulatory cell responses, and balancing Th1/Th2 cytokine profiles, phenolic acids influence immune cell activity (Liu et al., 2023). By reducing oxidative stress, their antioxidant qualities enhance these benefits by lowering chronic inflammation and fostering immunological homeostasis.

### 10.2 Cardiac and metabolic health

Phenolic acids are essential for enhancing metabolic and cardiovascular health due to their strong anti-inflammatory, lipid-lowering, and antioxidant qualities. These bioactive substances, which are typically present in whole grains, fruits, vegetables, and fermented foods, aid in lowering oxidative stress and inflammation, two main causes of metabolic syndrome and atherosclerosis. For instance, it has been proved that ferulic and caffeic acid improve endothelial function, avert LDL oxidation, and control blood lipid levels through enhancing HDL, lessening triglycerides, and total cholesterol content. Phenolic acids also impact glucose metabolism by increasing insulin sensitivity, monitoring glucose absorption, and inhibiting vital enzymes like α-glucosidase and -amylase (Gani et al., 2012). These insights make phenolic acids indispensable dietary constituents for the prevention and treatment of cardiometabolic disorders, also lessening obesity, type 2 diabetes, etc.

### 10.3 Neuroprotective and anti-aging potential

Clinical and preclinical studies have demonstrated the neuroprotective and anti-aging qualities of phenolic acids through a variety of mechanisms, including as antioxidant defense, anti-inflammatory effects, and the modification of neuronal signaling pathways. Compounds including gallic acid, ferulic acid, and caffeic acid have shown the potential to scavenge reactive oxygen species (ROS), protect against neuronal death, and reduce neuroinflammation by downregulating pro-inflammatory cytokines like TNF-α and IL-6. In preclinical models of Parkinson’s and Alzheimer’s diseases, phenolic acids have been demonstrated to enhance cognitive function, lessen the buildup of amyloid-β, and shield dopaminergic neurons (Caruso et al., 2022). In therapeutic settings, adding phenolic-rich extracts to older populations has been linked to increases in mood, memory, and cognitive resilience. Phenolic acids also enhance longevity-related pathways, such as Nrf2/ARE and sirtuins, which maintain cellular homeostasis and postpone senescence (Sharifi-Rad et al., 2023). These findings imply that regular phenolic acid intake, particularly from fermented or plant-based diets, may present viable methods for preventing neurological diseases and encouraging healthy aging.

## 11 Perspectives on technology and translation

### 11.1 Optimizing fermentation to increase the output of phenolic acid

Optimizing fermentation and increasing phenolic acid output requires a methodical strategy that involves modifying microbial strains, substrate composition, and process parameters to enhance the release and conversion of bound phenolics into their bioactive forms. Important elements include the use of specific microorganisms, such as *Lactobacillus*, *Bacillus*, and Aspergillus species, which are recognized for their enzymatic properties, especially in the action of esterases and decarboxylases, and the selection of high-phenolic substrates, such as whole grains, legumes, and fruit peels. Changing the fermentation conditions can have a substantial effect on microbial metabolism and phenolic acid output. Sequential fermentation or co-culturing strains can further boost yields by promoting synergistic enzymatic activity (Dissanayake et al., 2025). Additionally, pre-treatment methods like soaking, grinding, or enzymatic hydrolysis of the substrate can improve phenolic accessibility. For optimal outcomes, the protocol can be sensibly altered with the help of statistical methods like response surface methodology (RSM) and omics technologies. Conclusively, optimization of fermentation is important for producing functional foods with enhanced phenolic acid content and health benefits.

### 11.2 Encapsulation and delivery

Effective encapsulation and delivery strategies are essential to enhance the stability, bioavailability, and targeted release of phenolic acids, followed by antioxidants present in the functional foods and nutraceuticals. Because phenolic acids are recurrently prone to environmental circumstances like light, temperature, pH, and enzymatic deprivation result in decreased efficacy. To protect these materials during production, storage, and gastrointestinal transit, modern encapsulation strategies are utilized, like nanoemulsions, polymer-based particles, and so on (Esfanjani et al., 2018). These strategies have proven that phenolic acids attain their target region, like colon or certain tissues, in active levels by simplifying site-specific release. Subsequently, encapsulation helps the final product’s consumer appeal by enhancing solubility and helping to mask unpleasant flavors. Improving targeted functionality and augmenting the health benefits of phenolic acids whilst confirming regulatory compliance and product stability is possible through integrating advanced delivery systems responsive to pH (Rathee et al., 2023).

### 11.3 Regulatory considerations

Regulatory considerations about antioxidants are necessary to protect consumer care and end the propagation of deceptive information in food and nutraceutical labelling. Organizations like the European Food Safety Authority (EFSA) and the Food Safety and Standards Authority of India (FSSAI) have established rigorous protocols for verifying antioxidant rights. According to EFSA, all health claims, including those related to antioxidant activity, must be supported by solid scientific evidence demonstrating a cause-and-effect association in humans (Fernandes et al., 2024). Only medications that have quantifiable effects on oxidative stress biomarkers and clearly defined processes are eligible to make such claims. In a similar vein, the Food Safety and Standards Regulations, 2018 of the FSSAI only allow antioxidant claims that are supported by standardized testing methods and scientific validation. Both organisations highlight that claims such as “rich in antioxidants” or “helps combat oxidative stress” must be true and unambiguous, and that they must adhere to labelling rules, safety evaluations, and dosage constraints (Varghese and Ramamoorthy, 2023; Clemente-Suárez et al., 2025). These regulations are meant to safeguard consumers and ensure that products related to antioxidants have proven health benefits (Ochulor et al., 2022).

## 12 Conclusions and future perspectives

Phenolic acids, which are found in fermented foods, provide a good dietary choice for improving human health. Fermentation preserves and enhances food’s nutritional value while also increasing the bioavailability and bioactivity of phenolic acids through microbial biotransformation. They have anti-inflammatory, cardioprotective, neuroprotective, anti-diabetic, antioxidant, and anti-aging properties, among other health benefits. The synergistic interactions between phenolic acids, peptides, SCFAs during fermentation further boost their therapeutic potential. Preclinical and recent clinical studies suggest their effectiveness in lowering chronic illnesses such as metabolic syndromes, neurological disorders, and cardiovascular issues. Despite these encouraging results, more meticulously designed human studies are needed to validate dose-response relationships, bio-efficacy, and long-term safety. Subsequent investigations have to concentrate on comprehending the strain-specific microbial metabolism of phenolic acids, refining fermentation methods, and creating functional foods aimed at certain health objectives. To sum up, phenolic acid-enhanced fermented foods provide a socially acceptable, organic, and sustainable method of personalized nutrition and preventative healthcare.
